# Phosphatidylcholine’s influence on Dysmenorrhea: conclusive insights from Mendelian randomization analysis

**DOI:** 10.3389/fgene.2024.1404215

**Published:** 2024-09-23

**Authors:** Yuzheng Li, Shiyao Zhou, Yuchen Huang, Qiuhao Yu, Qibiao Wu

**Affiliations:** ^1^ Faculty of Chinese Medicine, and State Key Laboratory of Quality Research in Chinese Medicine, Macau University of Science and Technology, Macau, China; ^2^ Guangdong-Hong Kong-Macao Joint Laboratory for Contaminants Exposure and Health, Guangzhou, China; ^3^ Zhuhai MUST Science and Technology Research Institute, Zhuhai, Guangdong, China; ^4^ University Hospital, Macau University of Science and Technology Foundation, Macau, China

**Keywords:** phosphatidylcholine, Dysmenorrhea, Mendelian randomization (MR), causal analysis, GWAS

## Abstract

**Introduction:**

This study aimed to investigate the causal relationship between phosphatidylcholine (PC) levels and dysmenorrhea using Mendelian randomization (MR) analysis.

**Methods:**

We conducted a two-sample MR analysis using GWAS data on PC levels and dysmenorrhea. Single nucleotide polymorphisms (SNPs) associated with PC levels were used as instrumental variables. MR-Egger regression and inverse variance weighting (IVW) were used to estimate the causal effect of PC levels on dysmenorrhea. Sensitivity analyses were performed to assess the robustness of the results.

**Results:**

The IVW analysis revealed a significant positive association between higher PC levels and dysmenorrhea (OR: 1.533, 95% CI: 1.039–2.262, *P* = 0.031). The MR-Egger regression did not detect pleiotropy. Sensitivity analyses confirmed the robustness of the results.

**Conclusion:**

This study provides evidence suggesting a causal link between increased PC levels and dysmenorrhea. Further research is needed to understand the biological mechanisms underlying this relationship and to explore potential therapeutic implications.

## 1 Introduction

Menstrual discomfort, widely referred to as dysmenorrhea, represents a frequently encountered gynecological concern, afflicting between 45% and 72% of the female population worldwide and 43% to 93% of young women in their adolescence (Snipe et al., 2024) (Thomas and Magos, 2009). Research by Rafique N (Rafique and Al-Sheikh, 2018) shows that the global prevalence of primary dysmenorrhea (PD) is over 85.0%, especially among teenagers, where rates range from 56.1% to 93.0% (Hailemeskel et al., 2016). Common symptoms of dysmenorrhea include pain in the lower abdomen and sacral area, with some patients also experiencing headaches, back pain, vomiting, nausea, diarrhea, and sleep disturbances, which can significantly affect daily work and life when severe (Iacovides et al., 2014).

Dysmenorrhea is classified into two distinct types according to its origin: primary dysmenorrhea (PD) and secondary dysmenorrhea (SD). Typically, SD arises from associated pelvic disorders, including endometriosis, uterine fibroids, and pelvic inflammatory disease, with alleviation possible through addressing these foundational issues (Hu et al., 2020). Primary dysmenorrhea (PD) is characterized by recurrent abdominal pain concurrent with menstrual cycles in females whose reproductive organs are structurally and functionally normal. It is often manifested alongside other symptoms, including discomfort in the lower back, nausea, emesis, cephalalgia, and diarrheal symptoms. As outlined in the “Clinical Guidelines for Primary Dysmenorrhea,” established by the Canadian Association of Obstetrics and Gynecology in 2017, spasmodic pain located above the pubic area is a typical symptom of PD, which might also manifest as a continuous dull pain. This pain usually begins several hours before or after the start of menstrual bleeding and may last from 48 to 72 h (Burnett and Lemyre, 2017). The mechanism behind its occurrence is not fully understood, but studies have indicated that dysmenorrhea is related to the accumulation and release of prostaglandins and leukotrienes during menstruation (Harel, 2006; Bofill Rodriguez et al., 2019; Ge et al., 2011; Valentin et al., 2000).

Abnormal lipid levels are associated with various metabolic diseases, such as cardiovascular disease, diabetes, and obesity (Jia et al., 2024; Zhuang et al., 2023; Brydges et al., 2022; Kanno et al., 2014)^.^ Observational studies highlight a correlationbetween dysmenorrhea and lipid levels, pointing out that omega-3 polyunsaturated fatty acids (n-3 PUFA) have been associated with cognitive decline, a relationship ascribed to their anti-inflammatory attributes (Lin et al., 2022), and also related to the alleviation of dysmenorrhea symptoms (Proctor and Murphy, 2001). In recent years, lysoPC has attracted extensive attention of researchers. LysoPC is a metabolite of phosphatidylcholine (PC), which has been proved to play an important role in a variety of chronic inflammatory diseases, and regulate the function of immune cells by promoting the activity of inflammatory response, adhesion molecules and growth factors. In addition, lysoPC also promotes the synthesis of prostaglandins by inducing the expression of cyclooxygenase-2 (COX-2) in vascular endothelial cells, which is closely related to the pathogenesis of dysmenorrhea. Therefore, this study selected lysoPC as the research focus, not only to explore its potential biological mechanism in dysmenorrhea, but also to verify its causal relationship with dysmenorrhea by Mendelian randomized method.

Phosphatidylcholine (PC), particularly through its metabolite lysophosphatidylcholine (lysoPC), arises from the hydrolysis of PC in oxidized low-density lipoprotein (LDL), serving as a significant pro-inflammatory agent by facilitating inflammation via adhesion molecules, growth factors, monocytes, and macrophages. There is mounting evidence to indicate that lysoPC plays a role in modulating immune cell functionality, engaging in inflammatory activities, and participating in immune responses (Wu et al., 1999; Iwase et al., 2008). Therefore, lysoPC is regarded as a significant element in various chronic inflammatory conditions. Also, lysoPC has been found to induce cyclooxygenase-2 (COX-2) in vascular endothelial cells (Wu, 1998), a key enzyme in prostaglandin synthesis. These findings provide insights into how PC may be involved in the inflammatory mechanisms of dysmenorrhea. This study aims to investigate whether lipid levels such as PC affect dysmenorrhea. However, it is still unclear whether these associations have a causal relationship, and the biological mechanisms of this relationship are not fully elucidated. Additionally, potential confounding factors may affect the true association between phosphatidylcholine and dysmenorrhea, making clinical trial studies challenging, and lipid factors remain an insufficiently recognized target in clinical research on dysmenorrhea (Chen et al., 2024).

Mendelian randomization (MR) functions as a method for determining causal links, employing genetic variations as instrumental variables to evaluate the causal interplay between exposure factors and outcomes (Chen et al., 2024). Its primary benefit lies in minimizing the impact of confounding elements, thereby furnishing more dependable causal evidence. This investigation adopts the most recent genome-wide association study (GWAS) data available in 2023, applying MR to select specific single nucleotide polymorphisms (SNPs) closely linked with lipid levels for the analysis. SNPs such as rs56368075, rs1450746, rs1335712, and rs35610169 were utilized to examine the genetic underpinnings of the potential causal connection between phosphatidylcholine levels and dysmenorrhea, offering novel insights for the clinical management of dysmenorrhea.

## 2 Research methods

### 2.1 Research design

**FIGURE 1:**
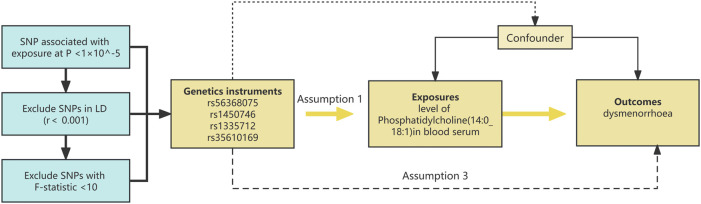
The diagrammatic representation illustrates the Mendelian randomization (MR) strategy employed to probe the putative causative link between phosphatidylcholine concentrations and dysmenorrhea. The MR assessments adhered to a triad of pivotal instrumental variable precepts: 1) The instrument must exhibit a substantial association with phosphatidylcholine levels (*p* < 1 × 10^−5). 2) The instrument should be devoid of associations with confounders that may affect the phosphatidylcholine-dysmenorrhea interplay. 3) The influence of the instrument on dysmenorrhea risk must be mediated exclusively by its impact on phosphatidylcholine levels. Instrumental variables, denoted by single nucleotide polymorphisms (SNPs), were employed in MR evaluations. The causality was gauged through methods such as inverse variance weighting (IVW) and the weighted median (WM) approach.

This research utilizes Mendelian randomization analysis, with genetic variants serving as instrumental variables. The comprehensive layout of the study is depicted in Figure 1, illustrating the flow chart of the overall design.

### 2.2 Data sources

This study retrieved data on dysmenorrhea and phosphatidylcholine as traits from the public GWAS database, GWAS Catalog. After the search, it selected the GWAS (genome-wide association studies) dataset on dysmenorrhea from the research by Longda Jiang et al. in November 2021 (Jiang et al., 2021) (https://www.ebi.ac.uk/gwas/studies/GCST90044465), and the GWAS dataset on phosphatidylcholine levels from the research by Ottensmann et al. (2023) (https://www.ebi.ac.uk/gwas/studies/GCST90277273). See Table 1 for details on the data sources.

**TABLE 1:** Table of raw data for phosphatidylcholine and dysmenorrhea studies from the GWAS database.

Trait	GWAS ID	Sample size	N case	N control	Population
Exposures level of Phosphatidylcholine (14:0_18:1) in blood serum	GCST90277273	7,064	NA	7,064	European
Dysmenorrhea	GCST90044465	247,540	413	247,127	European

### 2.3 GWAS data summary

Covering 7,174 Finnish participants, this study conducted univariate and multivariate GWAS analyses of 179 plasma lipid species, identifying 495 gene-trait associations involving eight new discoveries across 56 genetic loci. By precisely mapping variants with a high probability of causality and conducting PheWAS analysis among 377,277 participants in the FinnGen study, it explored the associations between these genes and diseases. Additionally, this study developed the fastGWA-GLMM tool suitable for large-scale biobank data, effectively accelerating the analysis of 2,989 binary traits in the UK Biobank data, demonstrating the potential to reveal associations between rare variants and complex traits. This GWAS analysis included 413 cases of dysmenorrhea and their phosphatidylcholine level study.

### 2.4 Mendelian randomization

Utilizing the “TwoSampleMR” package within R (software version 4.2.2), we engaged in Two-sample Mendelian Randomization (TSMR) analyses to identify SNPs linked to risk factors in GWAS pertinent to dysmenorrhea. We quantitatively analyzed SNPs using alleles’ frequencies and effect sizes, measured in beta coefficients and standard errors, to ensure precision in our causal inference. Phosphatidylcholine levels were quantified in micromoles per liter (μmol/L), providing a standardized measure for comparison. Throughout this process, we encountered instances of missing data, particularly in SNP genotype information. To address this, we adopted a comprehensive imputation strategy utilizing reference panels from the 1,000 Genomes Project, ensuring a robust dataset for analysis. This method allowed us to maintain statistical power while minimizing bias introduced by missing values.

We ensured the concordance of effect directions for associations between the risk factors and outcomes, rectifying palindromic SNPs with a minor allele frequency (MAF) below 0.3, and discarding those that did not comply. For each SNP, Wald estimates were calculated (by dividing the outcome estimate by the exposure estimate for the SNP) and synthesized using the inverse variance weighted (IVW) method under a random effects framework to derive estimates of causal effects of risk factors on dysmenorrhea. The IVW random effects model presupposes the absence of both directional and level pleiotropy.

To deepen the interpretation and strengthen the outcomes derived from inverse variance weighting (IVW), we employed additional sensitivity analyses including the Weighted Median estimator (WM) and MR-Egger regression techniques. These approaches help address underlying assumptions that are implicit in their application but not explicitly detailed. Specifically, the Weighted Median estimator assumes that less than half of the instrumental variables are subject to level pleiotropy, providing a robust estimate even if some of the instruments are invalid. Conversely, the MR-Egger regression is instrumental in addressing situations where a majority of the instrumental variables might be influenced by level pleiotropy, operating under the assumption that these influences are independent of the exposure variance. Though these methodologies inherently imply certain assumptions, including wider confidence intervals for MR-Egger, they are crucial for validating the resilience of our estimates against potential biases.

Given the extensive number of statistical tests performed in our analysis, we implemented the Benjamini-Hochberg procedure to adjust for multiple comparisons, targeting the false discovery rate (FDR). This approach was crucial in preserving the integrity of our findings, ensuring that the results presented were not merely artifacts of the large number of tests conducted but indicative of true associations. The adjustments made were based on FDR (*P* < 0.05) thresholds, which confirmed the robustness of our results, indicating a likely causal link between phosphatidylcholine levels and dysmenorrhea.

#### 2.4.1 Selection of instrumental variables

To obtain strongly associated instrumental variables (IVs), we screened for SNPs significantly associated with phosphatidylcholine levels (*P* < 1 × 10 ^-5) in R (software version 4.2.2) and used the “TwoSampleMR” package (version 0.5.7) to aggregate SNPs exhibiting linkage disequilibrium (r < 0.001) within a 10,000 kb window. This ensured no linkage disequilibrium between the included IVs, thereby maintaining the integrity of our analysis. The strength of IVs was determined by the magnitude and precision of the association between the genetic tool and the risk factor. An F statistic >10 was considered sufficient, calculated using the formula F = R (N-2)/(1-R ^2), where R represents the proportion of variance in EM and dysmenorrhea risk explained by IVs, and N is the sample size of the exposure GWAS. In our selection of SNPs as instrumental variables, we ensured the inclusion of relevant covariates such as age, sex, and body mass index (BMI) where data was available, using the same set of covariates for both exposure and outcome analyses to maintain consistency across our model. To ensure that the results were not affected by confounding factors, we excluded confounding factors such as BMI, tobacco and alcohol consumption, hormone levels, genetics, reproductive history, and autoimmune diseases, and ultimately found that rs1260326 was associated with BMI/alcohol consumption. Afterwards, MR analysis was conducted separately before and after excluding confounding factors.

#### 2.4.2 Causal estimation

MR Egger Regression: In our analysis, the Egger intercept test yielded a value of −0.0699 (Standard Error = 0.0559, *P*-value = 0.23), indicating the absence of significant pleiotropy. This result suggests that the causal estimates derived from our MR analysis are robust and unlikely to be confounded by pleiotropic effects of the instrumental variables on outcomes not mediated through phosphatidylcholine levels. In essence, this supports the integrity of our causal inference by affirming that the instrumental variables’ effect on dysmenorrhea is specifically through their association with phosphatidylcholine levels, rather than through other unrelated biological pathways.

Inverse Variance Weighting (IVW): The causal effect estimate obtained through the IVW method was β = 0.4274 with a 95% Confidence Interval of 0.0386–0.8163 (*P* = 0.0312). This finding underscores a statistically significant positive association between phosphatidylcholine levels and the risk of dysmenorrhea, suggesting that higher levels of phosphatidylcholine are potentially linked with an increased risk of developing dysmenorrhea. This method, assuming the absence of pleiotropy, provides a robust estimate of the causal relationship by leveraging the variance in instrumental variables to weight the causal effect estimation.

#### 2.4.3 Sensitivity analysis

The robustness of our causal inference was further validated through a sensitivity analysis using the Mendelian Randomization Pleiotropy Residual Sum and Outlier (MR-PRESSO) global test. The test yielded a *P*-value of 0.578, indicating no significant detection of outliers in our analysis. This result suggests a high degree of reliability in our causal inference results, affirming that the observed association between phosphatidylcholine levels and dysmenorrhea risk is consistent across different analytical methods and not driven by anomalous instrumental variable effects or outliers. This level of consistency bolsters the confidence in our findings and their potential implications for understanding dysmenorrhea’s pathophysiology.

#### 2.4.4 Reverse analysis data

Supplementary evidence reinforcing the potential influence of phosphatidylcholine concentrations on dysmenorrhea development was observed in our reverse analysis. Notably, the T allele of rs56368075 was found to have a significant positive association with dysmenorrhea risk (beta = 0.730055, *P* = 1.28731e-07), along with other SNPs such as rs1450746, rs1335712, and rs35610169, all showing significant positive correlations with phosphatidylcholine levels. These associations were highlighted in Table 2, providing additional layers of evidence supporting the causal link between elevated phosphatidylcholine levels and an increased risk of dysmenorrhea. The reverse analysis approach, examining the influence of genetic variants on phosphatidylcholine levels and subsequently on dysmenorrhea risk, lends further credence to our hypothesis and underscores the intricate genetic underpinnings of dysmenorrhea.

**TABLE 2:** Positive association between dysmenorrhea risk and phosphatidylcholine levels.

ID exposure	Rsid	Sample size	Beta	Pval	Popular
x2herE	rs56368075	247540	0.730055	1.29E-07	European
x2herE	rs1450746	245927	0.395661	2.54E-06	European
x2herE	rs1335712	247540	0.438931	1.32E-06	European
x2herE	rs35610169	238085	0.343187	2.73E-06	European

### 2.5 Registration

This study was retrospectively registered in the Open Science Framework (OSF) on 9 April 2024, under the DOI: https://doi.org/10.17605/OSF.IO/TDH68. The decision for late registration was driven by a commitment to enhance the transparency and reproducibility of our research findings. Initially, the importance and potential impact of pre-registration were not fully realized at the onset of our study. As the study progressed, it became evident that establishing a publicly accessible record of our research protocol, including hypotheses, methodology, and analysis plans, would significantly contribute to the integrity and validation of our research. Retrospective registration was chosen as a remedial step to document these details formally after recognizing the value of registration in fostering open science practices. This action underlines our dedication to uphold the principles of scientific transparency and allows for an explicit comparison between pre-specified plans and executed research, thereby providing context for interpreting our findings and enhancing the credibility of our study.

## 3 Results

To uphold the integrity of a Mendelian randomization study, three core principles must be observed: first, the genetic variant in question must exhibit a strong correlation with the risk factor (the relevance criterion); second, the genetic variant and the outcome should not share unaccounted confounders (the independence criterion); and third, the variant’s influence on the outcome should operate exclusively through the risk factor (the exclusion restriction criterion). Readers may consult the Mendelian Randomization dictionary for a more comprehensive understanding of the terminology applied herein. Only genetic variants adhering to these tripartite conditions are deemed suitable as instrumental variables. This Mendelian Randomization (MR) study is written in accordance with the STROBE-MR report (Skrivankova et al., 2021).

### 3.1 Selection and analysis of instrumental variables

Multiple single nucleotide polymorphisms (SNPs) served as instrumental variables (IVs) for assessing the influence of phosphatidylcholine on dysmenorrhea. Initial data analysis employed MR-Egger and inverse variance weighting (IVW) methodologies. The pleiotropy evaluation, indicated by the nonsignificant Egger regression intercept (*P* > 0.05), suggested an absence of notable pleiotropy concerns within the analysis of phosphatidylcholine’s relationship with dysmenorrhea, thereby affirming the reliability of the causal estimations.

### 3.2 Main analysis results

**FIGURE 2:**
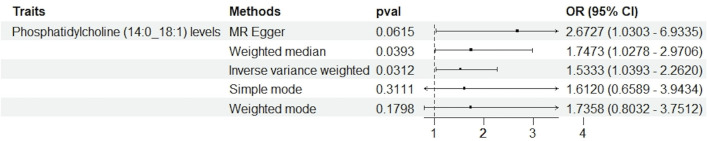
This figure illustrates the results of Mendelian randomization analyses assessing the association between phosphatidylcholine levels and dysmenorrhea. Various statistical methods were employed: MR Egger, Weighted Median, Inverse Variance Weighted (IVW), Simple Mode, and Weighted Mode. Each method evaluates the odds ratio (OR) with a 95% confidence interval (CI), depicted along the horizontal axis. The p-values are indicated for each analysis, with results suggesting varying degrees of association, highlighting the complexity and heterogeneity of the causal inference in this context.

The core findings from the Mendelian Randomization (MR) study, utilizing a range of MR techniques (such as MR Egger, weighted median, and inverse variance weighting), uniformly suggest a likely causal link between phosphatidylcholine concentrations and dysmenorrhea. Notably, the causal effect estimation derived via the inverse variance weighting (IVW) approach yielded an odds ratio (OR) of 1.533 (95% confidence interval: 1.039–2.262, *p* = 0.031). The forest plot illustrated in Figure 2 graphically conveys the causality, effect estimates, and their respective confidence intervals across various studies, affirming the association between elevated phosphatidylcholine levels and a heightened dysmenorrhea risk. After excluding confounding SNPs, we obtained similar results: the inverse variance weighting (IVW) approach yielded an odds ratio (OR) of 1.516 (95% confidence interval: 1.010–2.278, *P* = 0.044).

### 3.3 Sensitivity analysis

**FIGURE 3:**
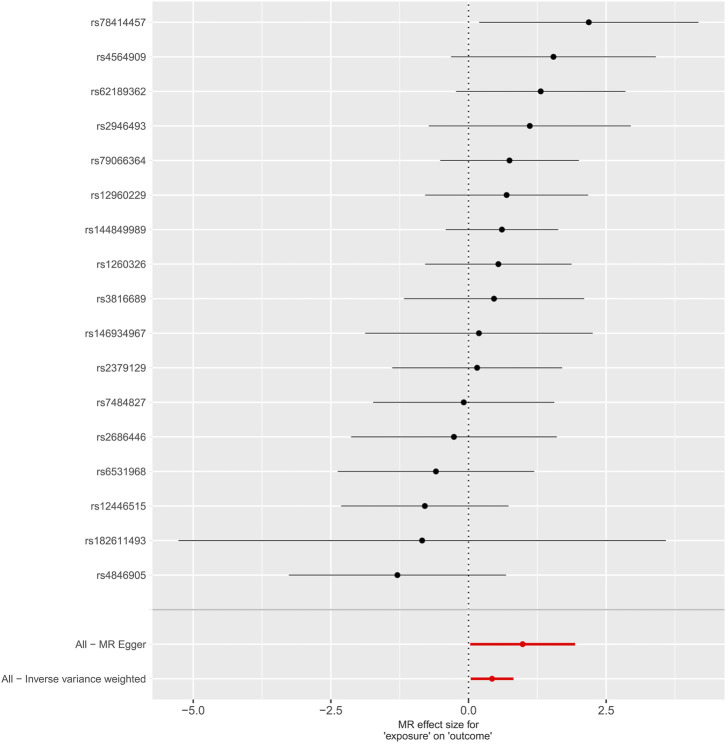
Graphical sensitivity analysis of the causal link between levels of phosphatidylcholine and dysmenorrhea risk.

Pleiotropy were assessed through the MR-PRESSO global test (*P* = 0.578), and no significant pleiotropy issues were detected, confirming the stability and reliability of the MR analysis results, as shown in the sensitivity analysis graph in Figure 3.

Heterogeneity was tested using Cochran’s Q test, and we found that its *P* values were all greater than 0.05 (*P* = 0.47).

### 3.4 Heterogeneity and pleiotropy analysis

**FIGURE 4:**
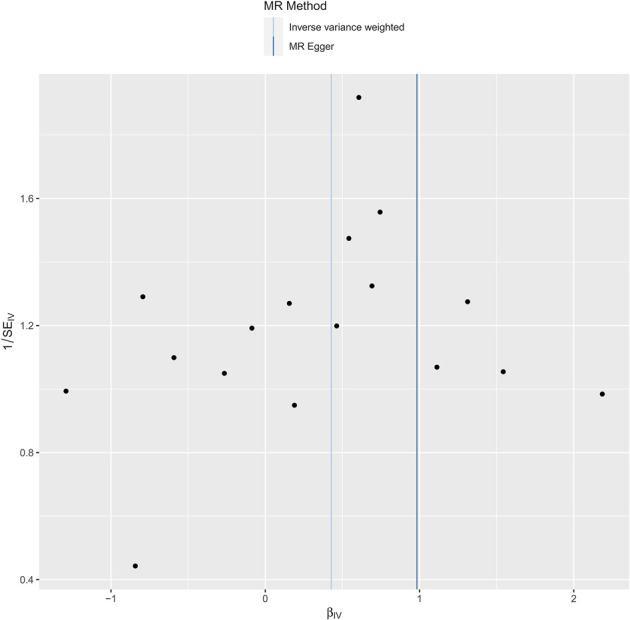
Funnel plot to test for bias in the effect of phosphatidylcholine levels on risk of dysmenorrhea in a Mendelian randomized trial.

**FIGURE 5:**
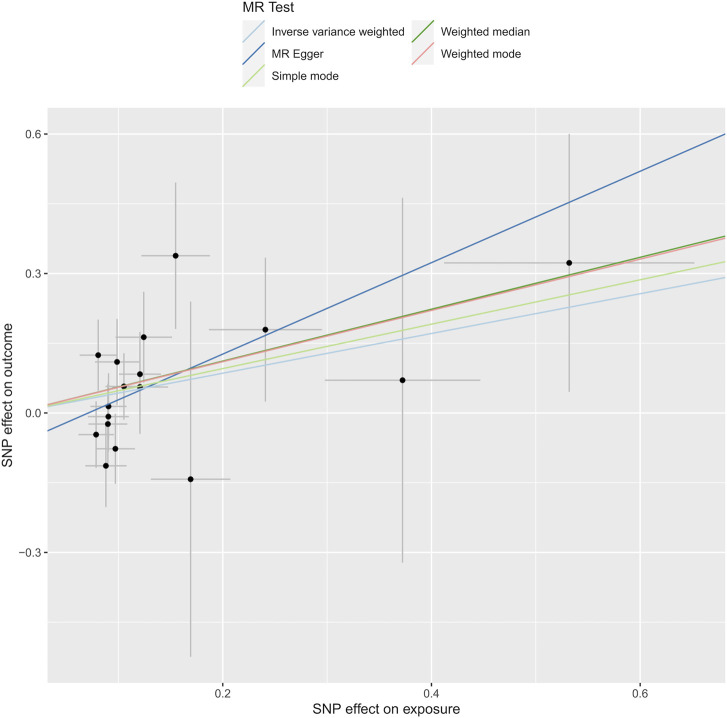
Depicts the analysis through Mendelian randomization of the causal linkage between levels of phosphatidylcholine and the susceptibility to dysmenorrhea.

In our research, we meticulously examined the potential for heterogeneity and pleiotropy. Utilizing Cochrane’s Q test and the MR-Egger intercept test, we identified no notable instances of heterogeneity or pleiotropy, thereby reinforcing the credibility of our causal conclusions. See Figures 4, 5. Heterogeneity was tested using Cochran’s Q test, and we found that its P values were all greater than 0.05 (*P* = 0.47).

## 4 Discussion

Employing data from the public GWAS Catalog and conducting a two-sample Mendelian Randomization analysis, this research investigated the causal link between Phosphatidylcholine and Dysmenorrhea. The results indicate a probable causal connection between Phosphatidylcholine and Dysmenorrhea, with an odds ratio of 1.533 (95% confidence interval: 1.039–2.262, *P* = 0.031). Additionally, the causal effect estimates and their confidence intervals from various studies indicate that an increase in Phosphatidylcholine levels is associated with a heightened risk of Dysmenorrhea.

The specific causes of primary dysmenorrhea are not fully understood, but it is commonly believed to be related to an increase in the frequency and intensity of uterinesmooth muscle contractions, which may be associated with the action of bioactive substances such as prostaglandins and progesterone in the body. Approximately 20%–25% of patients with Primary Dysmenorrhea (PD) (Oladosu et al., 2018; Lindh et al., 2012; Wang, 2019) who are treated using non-steroidal anti-inflammatory drugs (NSAIDs) demonstrate NSAID resistance and are susceptible to adverse effects, including gastrointestinal disturbances, endocrine metabolic irregularities, and water and sodium retention. Extended usage of oral contraceptives, calcium channel antagonists, and sedative antispasmodics has been linked to various adverse effects, including headaches, vertigo, reduced appetite, nausea, and emesis. A recent meta-analysis of 881 individuals (Snipe et al., 2024) with dysmenorrhea revealed that daily supplementation with 300–1800 mg of omega-3 long-chain polyunsaturated fatty acids for a period of 2 to 3 months is generally well-received and contributes to a decrease in both pain and the necessity for analgesics in women experiencing dysmenorrhea. In a study by Sadeghi et al. (2018), involving 100 college-aged women with PD, it was discovered that supplements of Omega-3 and vitamin E significantly reduced menstrual discomfort in comparison to a placebo, notably the combination of Vitamin E and omega-3 exhibited a pronounced effect on PD (*p* < 0.05). Additionally, research by Harel et al. (1996), which included 42 teenage sufferers of dysmenorrhea, demonstrated the advantageous impact of omega-3 fatty acid dietary supplementation on the symptoms associated with adolescent dysmenorrhea. In further research, Mehrpooya et al. (2017), carried out a randomized controlled trial to compare the efficacy of fish oil versus calcium supplementation in the management of PD, finding that omega-3 supplements surpassed calcium in effectiveness. These study results are consistent with the conclusions of this study, further verifying the causal relationship between phosphatidylcholine and dysmenorrhea.

In this study, we first revealed the possible causal relationship between PC and dysmenorrhea through MR analysis. Although the current literature on the direct relationship between PC and dysmenorrhea is relatively limited, we believe that the role of PC and its metabolite lysoPC in dysmenorrhea may be realized through the following mechanisms: first, lysoPC, as a metabolite of PC, is a known pro-inflammatory molecule. It promotes inflammation by activating monocytes and macrophages, and up regulating the expression of adhesion molecules and growth factors. This inflammatory response may be closely related to the pathophysiological mechanism of dysmenorrhea, especially by enhancing the inflammatory process in the endometrium, leading to excessive contraction and pain of uterine muscle. Secondly, lysoPC has been shown to induce the expression of COX-2 in vascular endothelial cells, which is a key enzyme in prostaglandin synthesis. Prostaglandins play an important role in dysmenorrhea. The increase of prostaglandins level is usually accompanied by more intense uterine contraction and more severe pain symptoms. Therefore, lysoPC may indirectly lead to the increase of prostaglandin levels by enhancing the expression of COX-2, thereby aggravating the symptoms of dysmenorrhea. In addition, PC and lysoprc may further aggravate dysmenorrhea by affecting the tension and contractility of uterine smooth muscle. These molecules may play a role in regulating the contraction of uterine smooth muscle through signaling pathways related to lipid metabolism. However, this hypothesis still needs further experimental research to verify. In conclusion, the findings of this study suggest that PC and its metabolite lysoPC may play a key role in the pathogenesis of dysmenorrhea. Future research should further explore the specific mechanism of these biomolecules in dysmenorrhea, and provide a theoretical basis for new treatment strategies.

At this stage, there are relatively few studies on the relationship between phosphatidylcholine levels and dysmenorrhea. This manuscript represents the inaugural application of Mendelian Randomization (MR) analysis to examine their causal linkage. Compared to small-scale studies with individual-level data processing, this study has the advantage of: 1) using large-scale GWAS data to study genetic data related to phosphatidylcholine levels and dysmenorrhea, which can minimize bias in determining causal effects; 2) Implementing Mendelian Randomization (MR) analysis to mitigate the effects of confounding variables and reverse causation, while affirming the solidity of our findings via sensitivity examinations; 3) Undertaking analyses of heterogeneity and pleiotropy using Cochrane’s Q test and the MR-Egger intercept test, observing no noteworthy heterogeneity or pleiotropy, thus bolstering the credibility of our causal deductions. However, this study has three limitations: 1) Since the GWAS data are from European populations, whether the conclusions are applicable to non-European populations needs further research; 2) Currently, there are no reports on the mechanism of how phosphatidylcholine levels relate to dysmenorrhea, so we cannot detail the mechanism between phosphatidylcholine levels and dysmenorrhea; 3) Employing genetic data precludes our ability to elucidate potential nonlinear dynamics or effects of stratification attributable to variables such as age, medication categories, and individual variances, all of which could contribute to heterogeneity within the research.

## 5 Conclusion

This study utilized Mendelian Randomization (MR) to explore the potential causal link between lipid concentrations, specifically phosphatidylcholine (PC) and its metabolite lysoPC, and dysmenorrhea. Our findings provide initial evidence that higher lipid levels may increase the likelihood of dysmenorrhea. These results suggest that targeting lipid metabolism could be a promising strategy for alleviating or preventing dysmenorrhea in clinical practice.

## Data Availability

The original contributions presented in the study are included in the article/supplementary material, further inquiries can be directed to the corresponding author.

## References

[B1] Bofill Rodriguez M. Lethaby A. Farquhar C. (2019). Non-steroidal anti-inflammatory drugs for heavy menstrual bleeding. Cochrane Database Syst. Rev. 9 (9), CD000400. 10.1002/14651858.CD000400.pub4 31535715 PMC6751587

[B2] Brydges C. R. Bhattacharyya S. Dehkordi S. M. Milaneschi Y. Penninx B. Jansen R. (2022). Metabolomic and inflammatory signatures of symptom dimensions in major depression. Brain Behav. Immun. 102, 42–52. 10.1016/j.bbi.2022.02.003 35131442 PMC9241382

[B3] Burnett M. Lemyre M. (2017). No. 345-Primary dysmenorrhea consensus guideline. J. Obstet. Gynaecol. Can. 39 (7), 585–595. 10.1016/j.jogc.2016.12.023 28625286

[B4] Chen J. Yu W. Guo T. Zhou Q. Niu P. Ye Y. (2024). Sleep characteristics and the risk of osteoarthritis: two-sample and multivariable Mendelian randomization analysis. China Tissue Eng. Res. 28 (32), 5203–5209.

[B5] Ge J. Haixia M. Xiaoli D. Yang Li (2011). Progress in clinical application of prostaglandins and isoprostanes. J. Gansu Coll. Tradit. Chin. Med. 28 (05), 65–68.

[B6] Hailemeskel S. Demissie A. Assefa N. (2016). Primary dysmenorrhea magnitude, associated risk factors, and its effect on academic performance: evidence from female university students in Ethiopia. Int. J. Womens Health 8, 489–496. 10.2147/IJWH.S112768 27695366 PMC5034908

[B7] Harel Z. (2006). Dysmenorrhea in adolescents and young adults: etiology and management. J. Pediatr. Adolesc. Gynecol. 19 (6), 363–371. 10.1016/j.jpag.2006.09.001 17174824

[B8] Harel Z. Biro F. M. Kottenhahn R. K. Rosenthal S. L. (1996). Supplementation with omega-3 polyunsaturated fatty acids in the management of dysmenorrhea in adolescents. Am. J. Obstet. Gynecol. 174 (4), 1335–1338. 10.1016/s0002-9378(96)70681-6 8623866

[B9] Hu Z. Tang L. Chen L. Kaminga A. C. Xu H. (2020). Prevalence and risk factors associated with primary dysmenorrhea among Chinese female university students: a cross-sectional study. J. Pediatr. Adolesc. Gynecol. 33 (1), 15–22. 10.1016/j.jpag.2019.09.004 31539615

[B10] Iacovides S. Avidon I. Bentley A. Baker F. C. (2014). Reduced quality of life when experiencing menstrual pain in women with primary dysmenorrhea. Acta Obstet. Gynecol. Scand. 93 (2), 213–217. 10.1111/aogs.12287 24266425

[B11] Iwase M. Sonoki K. Sasaki N. Ohdo S. Higuchi S. Hattori H. (2008). Lysophosphatidylcholine contents in plasma LDL in patients with type 2 diabetes mellitus: relation with lipoprotein-associated phospholipase A2 and effects of simvastatin treatment. Atherosclerosis 196 (2), 931–936. 10.1016/j.atherosclerosis.2007.02.012 17350631

[B12] Jia S. Chuanzheng W. Chengjin Q. Yuwei G. Aiyang W. Zhi L. (2024). Proteomics analysis of MRb_1 on skeletal muscle in type 2 diabetic mice induced by high-fat diet combined with streptozotocin. J. Jilin. Agric. Univ. 46 (3), 496–506. 10.13327/j.jjlau.2023.20333

[B13] Jiang L. Zheng Z. Fang H. Yang J. (2021). A generalized linear mixed model association tool for biobank-scale data. Nat. Genet. 53 (11), 1616–1621. 10.1038/s41588-021-00954-4 34737426

[B14] Kanno T. Jin Y. Nishizaki T. (2014). DL-/PO-phosphatidylcholine restores restraint stress-induced depression-related behaviors and spatial memory impairment. Behav. Pharmacol. 25 (5-6), 575–581. 10.1097/FBP.0000000000000063 25083573

[B15] Lin P. Y. Cheng C. Satyanarayanan S. K. Chiu L. T. Chien Y. C. Chuu C. P. (2022). Omega-3 fatty acids and blood-based biomarkers in Alzheimer's disease and mild cognitive impairment: a randomized placebo-controlled trial. Brain Behav. Immun. 99, 289–298. 10.1016/j.bbi.2021.10.014 34755655

[B16] Lindh I. Ellstrom A. A. Milsom I. (2012). The effect of combined oral contraceptives and age on dysmenorrhoea: an epidemiological study. Hum. Reprod. 27 (3), 676–682. 10.1093/humrep/der417 22252090

[B17] Mehrpooya M. Eshraghi A. Rabiee S. Larki-Harchegani A. Ataei S. (2017). Comparison the effect of fish-oil and calcium supplementation on treatment of primary dysmenorrhea. Rev. Recent Clin. Trials 12 (3), 148–153. 10.2174/1574887112666170328125529 28356030

[B18] Oladosu F. A. Tu F. F. Hellman K. M. (2018). Nonsteroidal antiinflammatory drug resistance in dysmenorrhea: epidemiology, causes, and treatment. Am. J. Obstet. Gynecol. 218 (4), 390–400. 10.1016/j.ajog.2017.08.108 28888592 PMC5839921

[B19] Ottensmann L. Tabassum R. Ruotsalainen S. E. Gerl M. J. Klose C. Widén E. (2023). Genome-wide association analysis of plasma lipidome identifies 495 genetic associations. Nat. Commun. 14 (1), 6934. 10.1038/s41467-023-42532-8 37907536 PMC10618167

[B20] Proctor M. L. Murphy P. A. (2001). Herbal and dietary therapies for primary and secondary dysmenorrhoea. Cochrane Database Syst. Rev. (3), CD002124. 10.1002/14651858.CD002124 11687013

[B21] Rafique N. Al-Sheikh M. H. (2018). Prevalence of primary dysmenorrhea and its relationship with body mass index. J. Obstet. Gynaecol. Res. 44 (9), 1773–1778. 10.1111/jog.13697 29974566

[B22] Sadeghi N. Paknezhad F. Rashidi Nooshabadi M. Kavianpour M. Jafari Rad S. Khadem Haghighian H. (2018). Vitamin E and fish oil, separately or in combination, on treatment of primary dysmenorrhea: a double-blind, randomized clinical trial. Gynecol. Endocrinol. 34 (9), 804–808. 10.1080/09513590.2018.1450377 29542390

[B23] Skrivankova V. W. Richmond R. C. Woolf B. A. R. Yarmolinsky J. Davies N. M. Swanson S. A. (2021). Strengthening the reporting of observational studies in epidemiology using mendelian randomization: the STROBE-MR statement. JAMA 326 (16), 1614–1621. 10.1001/jama.2021.18236 34698778

[B24] Snipe R. M. J. Brelis B. Kappas C. Young J. K. Eishold L. Chui J. M. (2024). Omega-3 long chain polyunsaturated fatty acids as a potential treatment for reducing dysmenorrhoea pain: systematic literature review and meta-analysis. Nutr. Diet. 81 (1), 94–106. 10.1111/1747-0080.12835 37545015

[B25] Thomas B. Magos A. (2009). Modern management of dysmenorrhoea. Trends Urol. Gynaecol. Sex. Health 14 (5), 25–29. 10.1002/tre.120

[B26] Valentin L. Sladkevicius P. Kindahl H. Broeders A. Marsal K. Melin P. (2000). Effects of a vasopressin antagonist in women with dysmenorrhea. Gynecol. Obstet. Invest 50 (3), 170–177. 10.1159/000010319 11014949

[B27] Wang X. (2019). Clinical efficacy observation of treating moderate to severe primary dysmenorrhea with medicine-separated moxibustion. China J. Tradit. Chin. Med. Pharm. 37 (07), 1582–1584.

[B28] Wu K. K. (1998). Injury-coupled induction of endothelial eNOS and COX-2 genes: a paradigm for thromboresistant gene therapy. Proc. Assoc. Am. Physicians 110 (3), 163–170. 9625523

[B29] Wu R. Svenungsson E. Gunnarsson I. Andersson B. Lundberg I. Schäfer Elinder L. (1999). Antibodies against lysophosphatidylcholine and oxidized LDL in patients with SLE. Lupus. 8 (2), 142–150. 10.1191/096120399678847434 10192509

[B30] Zhuang X. Wu L. Yin L. (2023). Progress in the study of body fat distribution and its relation to obesity-related chronic diseases. Jilin Med. J. 44 (07), 1932–1935.

